# Activated rate-response is associated with increased mortality risk in cardiac device carriers with acute heart failure

**DOI:** 10.1371/journal.pone.0302321

**Published:** 2024-04-18

**Authors:** Moritz T. Huttelmaier, Sascha Münsterer, Caroline Morbach, Floran Sahiti, Nina Scholz, Judith Albert, Alexander Gabel, Christiane Angermann, Georg Ertl, Stefan Frantz, Stefan Störk, Thomas H. Fischer

**Affiliations:** 1 Dept. of Internal Medicine I, University Hospital Würzburg, Würzburg, Germany; 2 Dept. Clinical Research & Epidemiology, Comprehensive Heart Failure Centre Würzburg, Würzburg, Germany; 3 Helmholtz Institute for RNA-based Infection Research (HIRI), Helmholtz Centre for Infection Research (HZI), Würzburg, Germany; 4 Infection Control and Antimicrobial Stewardship Unit, University Hospital Würzburg, Würzburg, Germany; Baruch Padeh Medical Center Poriya, ISRAEL

## Abstract

**Aims:**

This study investigated whether an activated R-mode in patients carrying a cardiac implantable electronic device (CIED) is associated with worse prognosis during and after an episode of acutely decompensated heart failure (AHF).

**Methods:**

Six hundred and twenty-three patients participating in an ongoing prospective cohort study that phenotypes and follows patients admitted for AHF were studied. We compared CIED carriers with activated R-mode stimulation (CIED-R) to CIED carriers not in R-mode (CIED-0) and patients without CIEDs (no-CIED). The independent impact of R-mode activation on 12-month all-cause death was examined using uni- and multivariable Cox proportional hazards regression taking into account potential confounders, and hazard ratios (HR) with their 95% confidence intervals (CI) were reported.

**Results:**

Mean heart rate on admission was lower in CIED-R (n = 37, 16% women) vs. CIED-0 (n = 64, 23% women) or no-CIED (n = 511, 43% women): 70 bpm vs. 80 bpm or 82 bpm; both p<0.001. In-hospital mortality was similar across groups, but age- and sex-adjusted all-cause 12-month mortality risk was differentially affected by R-mode activation; CIED-R vs. CIED-0: HR 2.44, 95%CI 1.25–4.74; CIED-R vs. no-CIED: HR 2.61, 95%CI 1.59–4.29. These effects persisted after multivariable adjustment for potential confounders. Within CIED-R, mortality risk was similar in patients with pacemakers vs. ICDs and in subgroups with left ventricular ejection fraction (LVEF) <50% vs. ≥50%.

**Conclusion:**

In patients admitted with AHF, R-mode stimulation was associated with a significantly increased 12-month mortality risk. Our findings shed new light on “admission heart rate” as a potentially treatable target in AHF. Our data are compatible with the concept that chronotropic incompetence contributes to an adverse outcome in these patients and may not be adequately treated through accelerometer-based R-mode stimulation.

## Introduction

Acute heart failure (AHF) is a life-threatening medical condition characterized by the rapid onset or deterioration of symptoms of heart failure (HF) [1]. Both heart rate and chronotropy affect outcome in chronic HF [2, 3]. In the randomized SHIFT trial, patients suffering from chronic HF with reduced left ventricular ejection fraction (LVEF ≤ 35%) whose resting heart rate was lowered by treatment with ivabradine experienced a marked reduction (-18%) of the combined endpoint hospital admissions for worsening HF or deaths due to HF [3]. Chronotropic incompetence, defined as an impaired increase of heart rate during physical activity or episodes of increased metabolic demand, is regarded a critical factor limiting cardiac output and exercise capacity in patients with HF [2]. As such, chronotropic incompetence predicted adverse events in chronic HF [4–6] and an increased mortality risk in the general population [7–10]. To treat symptomatic chronotropic incompetence, implantation of cardiac implantable electronic devices (CIED) operating in a rate-adaptive pacing mode (R-mode) is recommended [11]. However, accelerometers as the most frequently used sensors, depend on body movements and thus fail to modulate heart rate under circumstances of physical inactivity with high metabolic demand [12]. The latter, however, is a typical constellation encountered during acute cardiac decompensation. Accordingly, while a lower resting heart rate is associated with a more favourable prognosis in chronic HF [3], this may not apply to chronotropic incompetent patients experiencing an episode of acute decompensation. In these patients an unphysiologically low resting heart rate may be detrimental.

We therefore investigated if an activated R-mode affects prognosis in CIED carriers experiencing an episode of AHF.

## Methods

### Study design and patients

As part of an ongoing project of the Comprehensive Heart Failure Centre (CHFC) Würzburg, all patients admitted for acutely decompensated HF at the Department of Internal Medicine I of the University Hospital are identified and asked for participation in this single-centre prospective cohort study. Consenting patients are comprehensively and serially phenotyped to depict the natural course of disease throughout index hospitalization and up to five years after discharge. Written consent was obtained from all patients, and approval was granted by the Ethics Committee of the University of Würzburg (#55/14). The study is performed according to the principles of Good Clinical Practice and adheres to the Declaration of Helsinki and its later amendments.

### Clinical measurements and outcome ascertainment

Diagnosis of AHF was based on current guidelines by the physician on call and was verified considering all information obtained during the index hospitalisation. Exclusion criteria comprised cardiogenic shock, high output HF, and listing status for heart transplantation. At index hospitalisation, all patients underwent standardised examination and vital signs, medication at admission and discharge and secondary diagnoses were recorded. All patients received a 12-lead electrocardiogram (ECG) upon admission. The peripheral pulse rate, measured at least twice daily, was taken from the digital patient file. Routine echocardiography was performed by experienced sonographers, and LVEF was measured according to the highly standardized operating procedures installed at the Department of Internal Medicine I. Carriers of CIEDs at the timepoint of hospital admission were identified and the type of device and its pacing mode were obtained from patient records stored in the hospital information system. **S1 File** outlines our institutional approach to evaluation of chronotropic incompetence in CIED carriers. In-hospital worsening, defined as deteriorating HF symptoms, worsening laboratory values (renal function, liver function, natriuretic peptide) or deteriorating vital signs >24 hrs after admission were also monitored. Information on survival status and type and frequency of rehospitalizations were collected 6 and 12 months after the index hospitalization, either during outpatient visits at the CHFC Würzburg, by telephone follow-up, or based on information obtained from general practitioners, relatives, or registration authorities. The primary outcome measure was death from any cause at 12 months. Secondary outcomes analysed were in-hospital worsening, length of hospitalisation, in-hospital death, and rehospitalisation at 12 months after the index hospitalisation.

### Data analysis

Statistical analyses were performed using IBM SPSS Statistics software version 26. Data were described using mean (SD), median (quartiles), or count (percent), as appropriate. Depending on the presence of a CIED and the type of pacing mode, the study sample was grouped into patients carrying no cardiac device (no-CIED), devices with activated R-mode (CIED-R), and devices with inactivated R-mode stimulation (CIED-0). Based on the echocardiographically measured LVEF, patients were further studied in subgroups with LVEF <50% vs. LVEF ≥50%. We calculated the Charlson comorbidity index (CCI), a scoring tool measuring patients’ comorbidity disease status [13]. Equivalence doses for betablockers were calculated based on comparative effectiveness reported in guideline statements. Groups were compared using two-tailed t-test, ANOVA, Mann-Whitney U-test or Kruskal-Wallis test, or chi-square tests. Cox proportional hazard regression was used to identify univariable correlates of 12-month mortality risk, and hazard ratios (HR) with 95% confidence intervals (CI) were reported. The proportionality assumption was visually checked and found not to be violated. For multivariable models, age and sex were kept in fixed analyses, whereas the other potential confounders were selected using a backward likelihood ratio approach (p_out_ >0.1). Heart rate was not included into these models, because it might have depended on the activation of the sensor and was also presumed to be an indicator of chronotropic incompetence. No adjustment for multiple testing was introduced. All-cause mortality at 12 months across subgroups was modeled using Kaplan Meier (KM) curves and compared using the hazard ratio from respective crude Cox models. Statistical significance was assumed at a p-value <0.05.

## Results

### Baseline characteristics

We report data on the first 623 patients enrolled between Aug 2014 and Feb 2018. Characteristics at baseline of the total study sample and the different CIED subgroups are detailed in **Tables 1 and 2**. Mean age of the study population was 74 (11) years, and 61% were men. The median LVEF at admission was 51% (quartiles 32%, 59%). LVEF differed significantly between subgroups with CIED-0 showing lowest and no-CIED showing the highest LVEF values. The subgroups exhibiting LVEF <50% vs. LVEF ≥50% comprised 261 (47%) vs. 297 (53%) patients, respectively. Due to insufficient or lacking echocardiographic information, 65 patients could not be assigned to either LVEF subgroup. On admission, median NT-proBNP levels were 3522 pg/ml (1412–7818), and most patients (n = 581, 93%) were in NYHA functional class III or IV. Levels of N-terminal pro-hormone B-type natriuretic peptide (NT-proBNP) and distribution of New York Heart Association (NYHA) functional classes at admission were comparable between all subgroups. Levels of NT-proBNP were comparable between subgroups (**Tables 1 and 2**). *De novo* HF was detected in 20% (n = 120) of all patients. The proportion of *de novo* HF was highest in group no-CIED (23%), lowest in group CIED-0 (2%), and differed significantly between all subgroups. In the total sample, mean heart rate on admission was 81 (17) bpm and declined to 71 (14) bpm at discharge. 80% (n = 496) of all patients were treated with betablockers, 25% (n = 157) with glycosides, and 6% (n = 37) with amiodarone (**Tables 1 and 2**). Equivalence doses of betablockers were not different between subgroups. The median CCI was 3.0 (2.0, 4.0) in the total study sample and was comparable across all subgroups. Of the 112 patients (18%) carrying a CIED, 47 patients (8%) had a single chamber (1-CH-PM) or dual chamber pacemaker (2-CH-PM), 28 patients (5%) an implantable cardioverter defibrillator (ICD), and 37 patients (6%) a cardiac resynchronisation defibrillator system (CRT-D). No patients with His-, left bundle branch- or left bundle area-pacing were enrolled. Information about the underlying PM mode were available in 90% (n = 101) of patients carrying a CIED; patients with unknown PM mode (n = 11) were not considered in subgroup analyses. Within the CIED subgroup, 64 patients were programmed in VVI or DDD mode without rate adaption, whereas in 35 patients rate-adaptive pacing using an accelerometer was activated (VVIR, DDDR, DDDR-CRT). **Tables 3 and 4** shows respective patient characteristics according to the different types of CIEDs (PM vs. ICD vs. CRT-D).

**Table 1:** Baseline characteristics by presence of cardiac device and R-mode stimulation.

	All patients n = 623	CIED-R* n = 37	CIED-0* n = 64	no-CIED* n = 511	P value
** Age (years)**	74 (11)	74 (11)	72 (11)	74 (11)	0.15
** Male sex**	377 (60.5)	31 (83.8)	49 (76.6)	290 (56.8)	<0.001
** BMI (kg/m^2^)**	29.2 (6.5)	28.0 (4.8)	29.0 (5.5)	29.4 (6.8)	0.57
** NT-proBNP (pg/ml)**	3522 (1412–7818)	4117 (2000–7612)	4166 (2465–10139)	3291 (1256–7614)	0.05
** Heart rate, at admission (bpm)**	81 (17)	70 (11)	80 (16)	82 (17)	<0.001
** Heart rate, at discharge (bpm)**	71 (14)	66 (12)	71 (14)	72 (14)	0.026
** eGFR, at admission (ml/min/1.73 m^2^)**	47 (33–65)	40 (27–51)	40 (28–58)	49 (34–66)	0.020
** eGFR, at discharge (ml/min/1.73 m^2^)**	46 (32–64)	38 (27–52)	49 (28–60)	47 (32–65)	0.034
**Heart failure subtype by LVEF^+^**					
** LVEF (%)**	51 (32–59)	45 (28–55)	28 (20–48)	53 (35–60)	<0.001
** LVEF <50%**	261 (46.8)	20 (57.1)	44 (78.6)	191 (41.5)	<0.001
** LVEF ≥50%**	297 (53.2)	15 (42.9)	12 (21.4)	269 (58.5)	
**Clinical presentation, at admission**					
***De novo* heart failure**	120 (19.6)	2 (5.4)	1 (1.6)	117 (22.9)	<0.001
** Chronic heart failure**	492 (80.4)	35 (94.6)	63 (98.4)	394 (77.1)	
** NYHA functional class II**	31 (5.0)	1 (2.8)	4 (6.3)	24 (4.8)	0.81
** NYHA functional class III**	280 (44.9)	17 (47.2)	32 (50.8)	226 (45.0)	
** NYHA functional class IV**	301 (48.3)	18 (50.0)	27 (42.9)	252 (50.2)	
**Comorbidities**					
** Diabetes mellitus**	264 (42.4)	17 (48.6)	27 (43.5)	214 (42.8)	0.82
** Arterial hypertension**	517 (83.0)	27 (77.1)	58 (90.6)	424 (84.8)	0.20
**Atrial fibrillation^#^**	353 (56.7)	30 (81.1)	40 (62.5)	279 (54.6)	0.004
**Coronary artery disease**	285 (45.7)	15 (40.5)	45 (70.3)	221 (43.2)	<0.001
** Charlson comorbidity index**	3.0 (2.0–4.0)	3.0 (2.0–3.5)	3.0 (2.0–4.0)	3.0 (2.0–4.0)	0.29
**Heart failure medication**					
** Betablocker**	496 (79.6)	28 (75.7)	59 (92.2)	409 (80.0)	0.084
** ACEi or ARB or ARNI**	442 (70.9)	20 (54.1)	43 (67.2)	379 (74.2)	0.019
** MRA**	226 (36.3)	20 (54.1)	35 (54.7)	171 (33.5)	<0.001
** Diuretics**	560 (89.9)	34 (91.9)	62 (96.9)	464 (90.8)	1.0
**Bradycardia inducing medication^§^**					
** Glycoside**	157 (25.2)	11 (29.7)	22 (34.4)	118 (23.1)	0.11
** Ivabradine**	30 (4.8)	2 (5.4)	10 (15.6)	17 (3.3)	0.001
** Amiodarone**	37 (5.9)	9 (24.3)	11 (17.2)	17 (3.3)	<0.001
**Cardiac device**					
** no-CIED**	511 (82.0)	--	--	--	--
** PM**	47 (7.5)	21 (56.8)	21 (32.8)	--	
** ICD**	28 (4.5)	4 (10.8)	20 (31.3)	--	
** CRT-D**	37 (5.9)	12 (32.4)	23 (35.9)	--	

Data are n (%), mean (SD), or median (quartiles). P-values refer to overall test across subgroups (chi-square-test or Kruskal-Wallis test, as appropriate).

*CIED (n = 11) with unknown PM mode were not considered in subgroups.

^+^LVEF was measured in 558 patients.

^#^Prevalence of any type of atrial fibrillation, assessed at discharge.

^§^Assessed at discharge.

CIED = cardiac implantable electronic device, R = with rate response sensor (accelerometer), 0 = without rate response sensor (accelerometer), no-CIED = no CIED implanted, BMI = body mass index, NT-proBNP = N-terminal pro-hormone B-type natriuretic peptide, eGFR = estimated glomerular filtration rate, LVEF = left ventricular ejection fraction, ACEi = angiotensin converting enzyme inhibitor, ARB = angiotensin II receptor type 1 blocker, ARNI = angiotensin receptor neprilysin inhibitor, MRA = mineralocorticoid receptor antagonist, PM = pacemaker, ICD = implantable cardioverter defibrillator, CRT-D = cardiac resynchronisation device with defibrillator.

**Table 2:** Clinical events during hospitalisation and post-discharge by presence of cardiac device and R-mode stimulation.

	All patients n = 623	CIED-R* n = 37	CIED-0* n = 64	no-CIED* n = 511	P value
**Length of hospitalisation (days)**	10 (7–14)	11 (6–20)	11 (7–15)	10 (7–14)	0.23
**In-hospital worsening**	412 (66.1)	27 (73.0)	38 (59.4)	340 (66.7)	0.35
**In-hospital death, n (%)**	14 (2.2)	1 (2.7)	0 (0.0)	12 (2.3)	0.49
**Rehospitalisation within 12 months**	422 (67.7)	30 (81.1)	49 (76.6)	334 (65.4)	0.037
**Death of any cause within 12 months**	161 (25.8)	19 (51.4)	17 (26.6)	122 (23.9)	0.001

Data are n (%) or median (quartiles). P-values refer to overall test across subgroups (chi-square test or Kruskal-Wallis test, as appropriate).

Abbreviations as in Table 1. *CIED (n = 11) with unknown PM mode were not considered in subgroups.

**Table 3:** Characteristics by type of cardiac device.

	No-CIED n = 511	PM n = 47	ICD n = 28	CRT-D n = 37	P value
** Age (years)**	74 (11)	79 (9)	70 (9)	67 (12)	<0.001
** Male sex**	290 (56.8)	33 (70.2)	23 (82.1)	31 (83.8)	<0.001
** BMI (kg/m^2^)**	29.4 (6.8)	27.9 (5.4)	28.2 (4.1)	29.7 (5.6)	0.46
** NT-proBNP (pg/ml)**	3522 (1412–7818)	3291 (1256–7614)	3600 (2181–8439)	5003 (2340–9801)	0.081
** Heart rate, at admission (bpm)**	82 (17)	78 (18)	81 (14)	70 (11)	0.013
** Heart rate, at discharge (bpm)**	72 (14)	71 (18)	71 (9)	66 (12)	0.30
** eGFR, at admission (ml/min/1.73m^2^)**	47 (33–65)	49 (34–66)	37 (27–54)	48 (30–60)	<0.001
** eGFR, at discharge (ml/min/1.73m^2^)**	46 (32–64)	47 (32–65)	38 (30–53)	48 (28–69)	0.018
Heart failure subtype by LVEF*					
** LVEF (%)**	53 (35–60)	51.0 (41–57)	25 (20–35)	24 (17–29)	<0.001
** LVEF <50%**	198 (41.5)	18 (41.9)	24 (96.0)	28 (93.3)	<0.001
** LVEF ≥50%**	269 (58.5)	25 (58.1)	1 (4.0)	2 (6.7)	
**Clinical presentation, at admission**					
***De novo* heart failure**	117 (22.9)	5 (10.6)	0	0	<0.001
** Chronic heart failure**	394 (77.1)	42 (89.4)	28 (100)	37 (100)	
** NYHA functional class II**	24 (4.7)	0	5 (17.9)	2 (5.4)	0.026
** NYHA functional class III**	226 (44.2)	23 (48.9)	15 (53.6)	16 (43.2)	
** NYHA functional class IV**	252 (49.3)	24 (51.1)	8 (28.6)	17 (45.9)	
**Comorbidities**					
** Diabetes mellitus**	214 (41.9)	21 (44.7)	10 (35.7)	19 (51.4)	0.64
** Arterial hypertension**	424 (83.0)	41 (87.2)	26 (92.9)	26 (70.3)	0.06
**Atrial fibrillation^#^**	279 (54.6)	32 (68.1)	19 (67.9)	23 (62.2)	0.18
** Coronary artery disease**	221 (43.2)	27 (57.4)	20 (71.4)	17 (45.9)	0.009
** Charlson comorbidity index**	3.0 (2.0–4.0)	3.0 (2.0–4.0)	3.0 (2.0–3.0)	3.0 (2.0–4.0)	0.169
**Heart failure medication**					
** Betablocker**	409 (80.0)	40 (85.1)	23 (82.1)	33 (89.2)	0.55
** ACEi, or ARB or ARNI**	379 (74.2)	26 (55.3)	21 (75.0)	22 (59.5)	0.016
** MRA**	171 (33.5)	17 (36.2)	20 (71.4)	23 (62.2)	<0.001
** Diuretics**	464 (90.8)	43 (91.5)	26 (92.9)	36 (97.3)	1.0
**Bradycardia inducing medication^§^**					
** Glycoside**	118 (23.1)	12 (25.5)	14 (50.0)	13 (35.1)	0.007
** Ivabradine**	17 (3.3)	1 (2.1)	5 (17.9)	7 (18.9)	<0.001
** Amiodarone**	17 (3.3.)	2 (4.3)	5 (17.9)	13 (35.1)	<0.001

Data are n (%), mean (SD), or median (quartiles). P-values refer to overall tests across subgroups (chi-square-test or Kruskal-Wallis test, as appropriate).

*LVEF was measured in 558 patients.

^#^Prevalence of any type of atrial fibrillation, assessed at discharge.

^§^Assessed at discharge.

No-CIED = no cardiac implantable electronic device (CIED), PM = pacemaker, ICD = implantable cardioverter defibrillator, CRT = cardiac resynchronisation device with defibrillator, BMI = body mass index, NT-proBNP = N-terminal pro-hormone B-type natriuretic peptide, eGFR = estimated glomerular filtration rate, LVEF = left ventricular ejection fraction, ACEi = angiotensin converting enzyme inhibitor, ARB = angiotensin II receptor type 1 blocker, ARNI = angiotensin receptor neprilysin inhibitor, MRA = mineralocorticoid receptor antagonist.

**Table 4:** Clinical events during hospitalisation and post-discharge by type of cardiac device.

	No-CIED n = 511	PM n = 47	ICD n = 28	CRT-D n = 37	P value
Length of hospitalisation (days)	10 (7–4)	10 (7–16)	8.5 (7–14)	13 (9–22)	0.026
Intra-hospital worsening	340 (66.7)	29 (61.7)	14 (50.0)	29 (78.4)	0.10
In-hospital death, n (%)	12 (2.3)	2 (4.3)	0 (0.0)	0 (0.0)	0.42
Rehospitalisation within 12 months	334 (65.4)	33 (70.2)	23 (82.1)	30 (81.1)	0.07
Death of any cause within 12 months	122 (23.9)	15 (31.9)	6 (21.4)	18 (48.6)	0.007

Data are n (%) or median (quartiles).

P-values refer to overall test across subgroups (chi-square-test or Kruskal-Wallis test, as appropriate).

No-CIED = no cardiac implantable electronic device (CIED), PM = pacemaker, ICD = implantable cardioverter defibrillator, CRT = cardiac resynchronisation device with defibrillator.

### Clinical events during index hospitalisation and follow-up

The median length of index hospitalization was 10 (7, 14) days and was comparable across subgroups (**Tables 1 and 2**). An episode of in-hospital worsening occurred in 66% of all patients, and 2.2% of the overall sample died during the index hospitalization. No significant differences were found between subgroups in terms of percentage of in-hospital worsening or in-hospital mortality. Within 12 months of follow-up, 68% of all patients were readmitted to hospital, and 26% of patients died. All-cause-mortality at 12 months differed significantly between groups no-CIED (24%), CIED-0 (27%) and CIED-R (51%; p = 0.001). The in-hospital and post-discharge course of all CIED subgroups is also shown in **Table 2**. The occurrence of clinical events depending on the type of CIED (PM, ICD, CRT-D) is presented in **Table 4**.

### Heart rate pattern during index hospitalisation and prognostic impact

**Fig 1:**
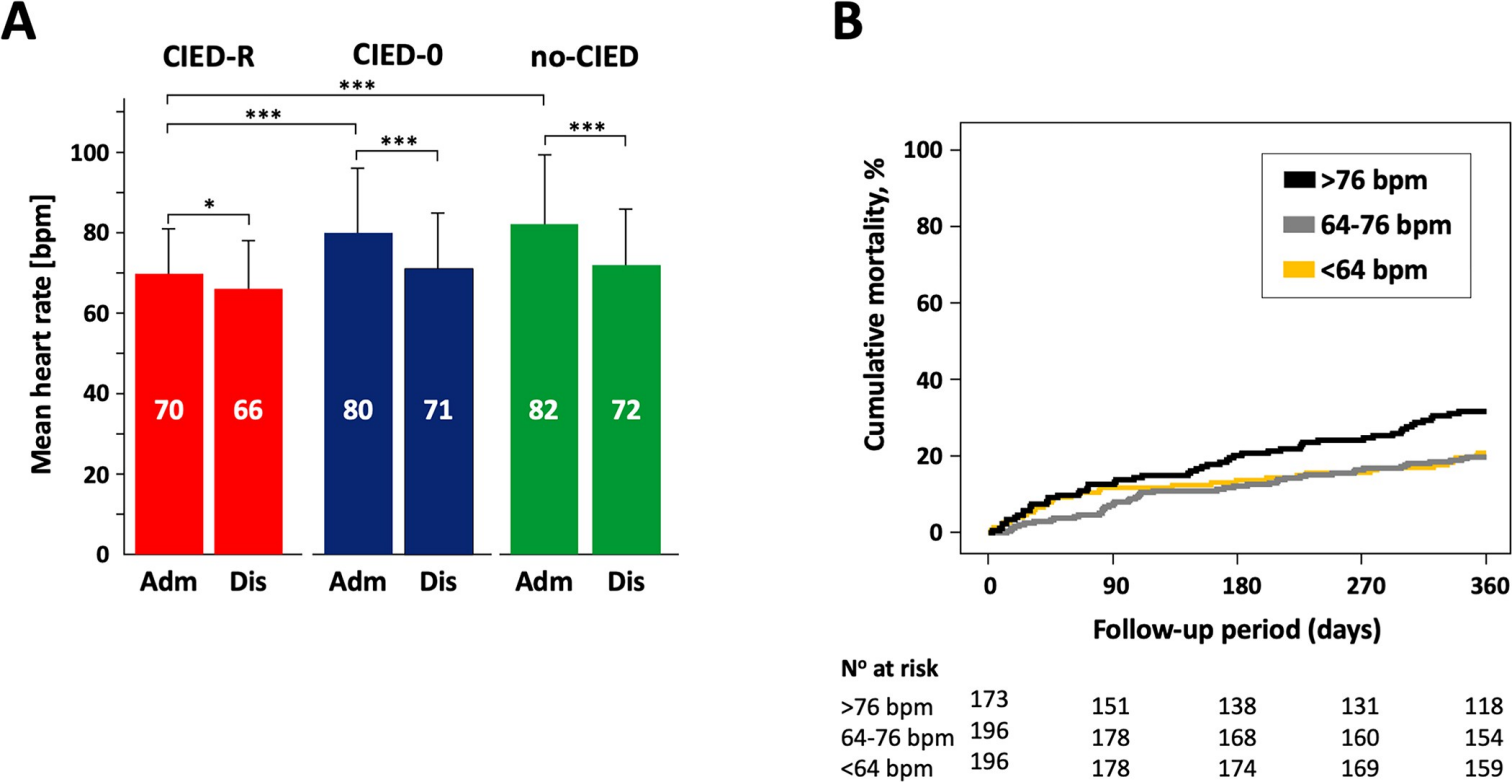
Changes in resting heart rate during index hospitalization by CIED group. **A)** Comparison of group CIED-R (CIED with rate adaptive pacing) vs CIED-0 (CIED without rate adaptive pacing) vs no-CIED (no CIED) from admission (ADM) to discharge (Dis). Data are mean (SD). *p<0.05, **p<0.01, ***p<0.001. **B)** All-cause death risk during the 12-month follow-up period by tertiles of resting heart rate at discharge from hospital after exclusion of patients with an activated R-mode (CIED-R), adjusted for age and sex. Tertile 1: < 64 bpm, tertile 2: 64–76 bpm, tertile 3: >76 bpm.

**Fig 2:**
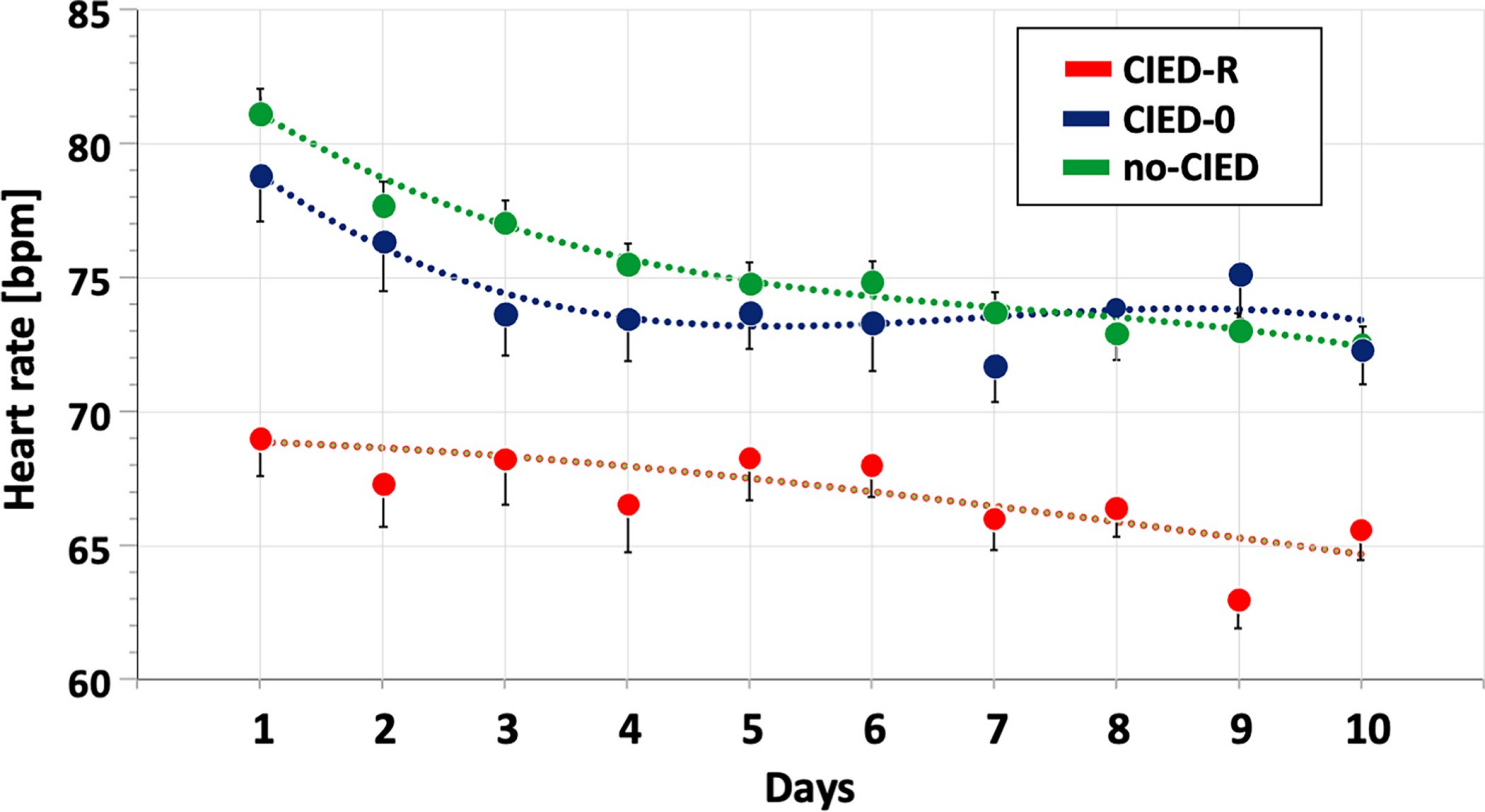
Longitudinal analysis of heart rate from day 1 through day 10 at index hospitalization. Comparison of daily mean heart rate from day 1 through day 10 during index hospitalization between groups CIED-R (CIED with rate adaptive pacing), CIED-0 (CIED without rate adaptive pacing) and no-CIED (no CIED).

Mean heart rate on admission was significantly lower in group CIED-R vs. no-CIED or CIED-0: 70 bpm vs. 82 bpm or 80 bpm, both p<0.001 (**Fig 1A**). Whereas all groups experienced a decrease in heart rate between admission and discharge, the reduction in group CIED-R was about 50% smaller compared to CIED-0 and no-CIED (**Fig 1A**). Further, as illustrated in **Fig 2**, heart rate in group CIED-R remained significantly lower throughout the entire period of the first 10 days of hospitalisation.

We assessed the variables associated with length of index hospitalisation, frequency of episodes of in-hospital worsening and all-cause 12-month mortality in tertiles of heart rate on admission and at discharge, respectively. Overall, heart rate on admission had no impact on duration of index hospitalization, frequency of in-hospital worsening, or death. However, after excluding CIED-carriers with an activated R-mode (CIED-R), patients with the highest discharge heart rate (>76 bpm) showed a significantly increased all-cause mortality risk compared to patients with a discharge heart rate in the lowest tertile (<64 bpm): HR 1.53, 95%CI 1.03–2.30, p = 0.04, adjusted for age and sex. This effect persisted after adjustment for bradycardia inducing medication and CCI. The respective Kaplan-Meier plot is displayed in **Fig 1B**.

### Prognostic impact of type of device and activated rate response

#### CIED-R vs. no-CIED

**Fig 3:**
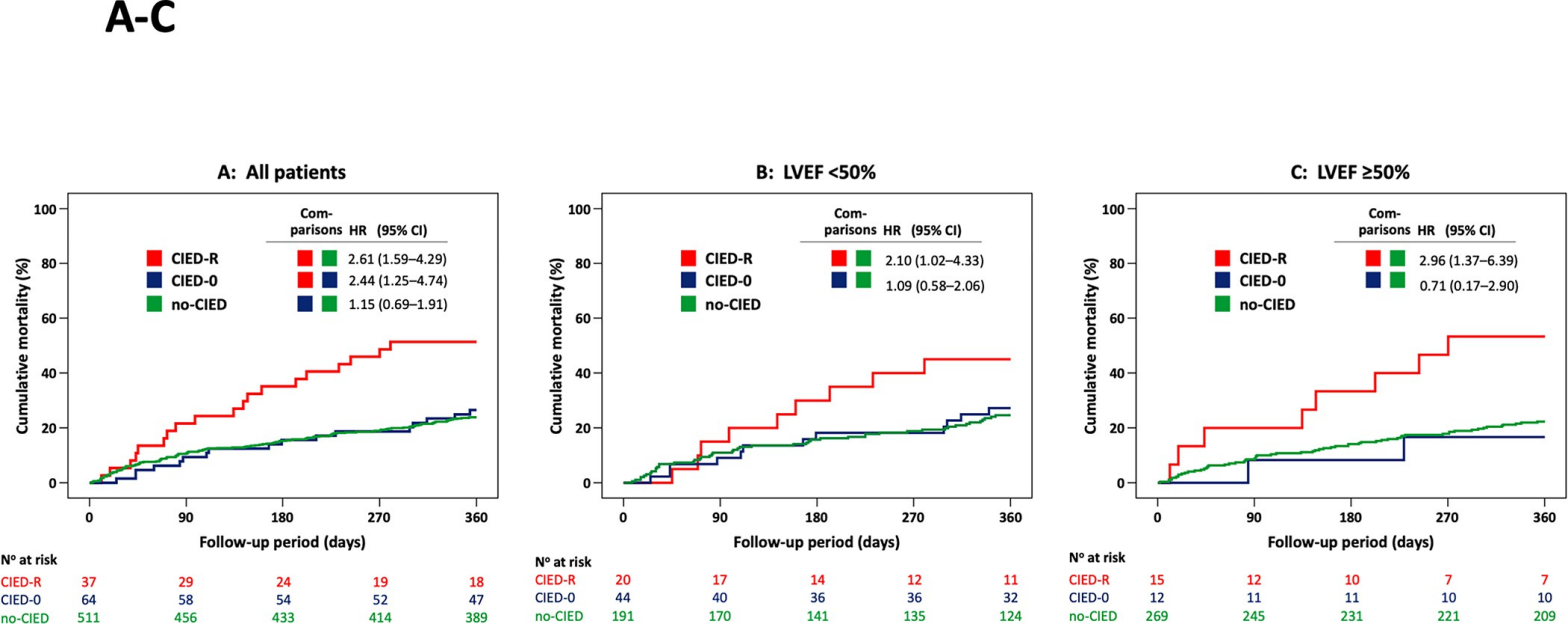
Mortality risk (Kaplan Meier plot) during the 12-month follow-up period by CIED group and type of heart failure. **A)** Study population. Comparison of group CIED-R (CIED with rate adaptive pacing), CIED-0 (CIED w/o rate adaptive pacing) and no-CIED (no CIED). **B)** LVEF <50%. Comparison of group CIED-R (CIED with rate adaptive pacing) vs CIED-0 (CIED without rate adaptive pacing) vs no-CIED (no CIED). **C)** LVEF ≥50%. Comparison of group CIED-R vs CIED-0 vs no-CIED. Hazard ratio (HR) with 95% confidence interval (CI) from Cox proportional hazards regression, adjusted for age and sex.

Mortality at 12 months was higher in group CIED-R compared to group no-CIED (unadjusted HR 2.61, 95%CI 1.61–4.23, p = 0.001; **Fig 3A**). Remarkably, mortality of CIED-R patients was similar amongst carriers of a PM-R vs. ICD-R (unadjusted HR 1.20, 95% CI 0.49–2.95, p = 0.70) and in subgroups with LVEF below vs. above 50% (unadjusted HR 1.10, 95% CI 0.79–1.53, p = 0.59). Regardless of LVEF, patients in group CIED-R had an increased mortality risk compared to no-CIED patients (**Fig 3B and 3C**).

#### CIED-0 vs. no-CIED

Mortality at 12 months was similar in group CIED-0 and no-CIED: unadjusted HR 1.10, 95% CI 0.66–1.82, p = 0.72 (**Fig 3A**). When comparing patients carrying a PM without activated R-mode (PM-0) and patients carrying a defibrillator without activated R-mode (ICD-0), also no differences were seen: unadjusted HR 1.65, 95% CI 0.54–5.05, p = 0.38. Further, mortality risk in CIED-0 patients was independent from LVEF (**Fig 3B and 3C**).

#### CIED-R vs. CIED-0

**Fig 4:**
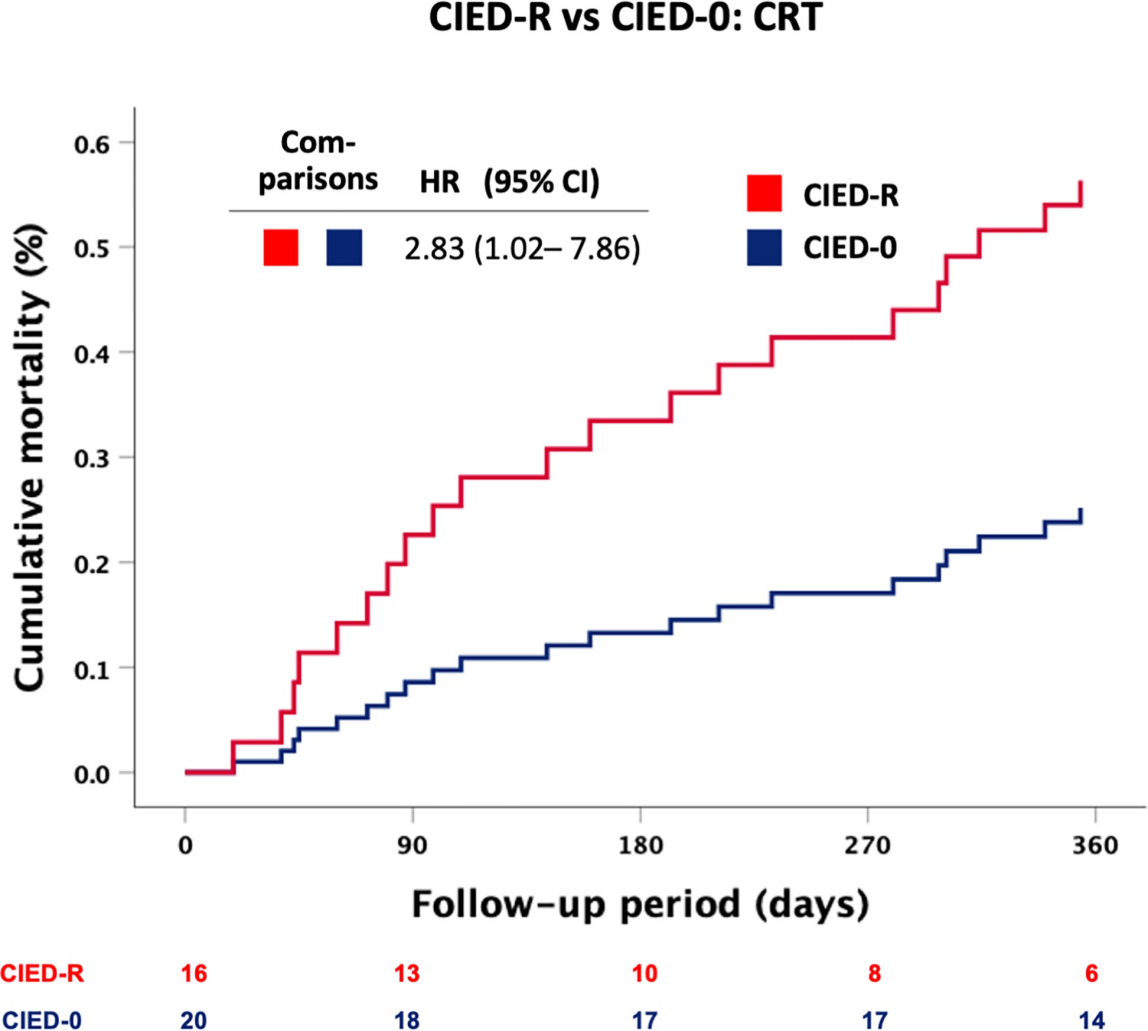
Mortality risk (Kaplan Meier plot) during the 12-month follow-up period in the CRT subgroup. Comparison of group CIED-R (CRT with rate-adaptive pacing) to CIED-0 (CRT without rate adaptive pacing). Unadjusted Hazard ratio (HR) with 95% confidence interval (CI) from Cox proportional hazards regression.

Consistent with the preceding comparisons, mortality at 12 months was higher in group CIED-R compared to CIED-0: unadjusted HR 2.43, 95%CI 1.26–4.68, p = 0.01 **(Fig 3A)**. Whereas this difference was also apparent in the subgroup with LVEF ≥50% (**Fig 3C**), it was absent in the subgroup with LVEF<50% (**Fig 3B**). Further, the effect persisted i) after exclusion of patients with single chamber devices programmed in VVIR-mode, who exhibit the highest risk for a R-mode induced increment in RV pacing burden (unadjusted HR 2.86, 95%CI 1.18–6.92, p = 0.02) and ii) when exclusively assessing CIEDs with biventricular pacing (CRT subgroup): unadjusted HR 2.83, 95% CI 1.02–7.86, p = 0.045 **(Fig 4).**

Amongst univariate predictors of mortality risk, strong associations were found for NT-proBNP levels, atrial fibrillation (AF), CCI, and HF occurring *de novo* (**Table 5**). When adjusting for age and sex, the HR of CIED-R was 2.61 (95% CI 1.59–4.29, p<0.001) compared to group no-CIED, and 2.44 (95%CI 1.25–4.74, p = 0.009) compared to group CIED-0. These associations were not significantly altered by consideration of all other potential confounders in a fully adjusted model (**Table 5**).

**Table 5:** Uni- and multivariable correlates of 12-month mortality risk.

	Univariable analysis		Multivariable analysis	
Covariate	HR (95% CI)	P value	HR (95% CI)	P value
**Age (years)**	1.02 (1.01–10.4)	0.011	1.03 (1.01–1.05)	0.013
**Male sex**	1.03 (0.75–1.42)	0.84	1.11 (0.77–1.60)	0.57
**Body mass index, per kg/m^2^**	0.97 (0.94–1.00)	0.032	--	--
**LVEF, per 5%**	1.00 (0.98–1.03)	0.42	--	--
**NT-proBNP (pg/ml), per tertile ^1^**	2.04 (1.63–2.54)	<0.001	1.85 (1.47–2.34)	<0.001
**eGFR at discharge, per 5 ml/min./1.73m^2^**	0.93 (0.89–0.96)	<0.001	--	--
**Diabetes mellitus, yes vs no**	1.13 (0.83–1.55)	0.45	--	--
**Arterial hypertension, yes vs no**	1.03 (0.66–1.60)	0.91	--	--
Atrial fibrillation**, yes vs no	1.49 (1.08–2.05)	0.016	--	--
**Coronary artery disease, yes vs no**	0.95 (0.70–1.30)	0.75	--	--
**Charlson comorbidity index, per tertile ^2^**	1.45 (1.16–1.80)	0.001	--	--
**Type of heart failure**				
** LVEF ≥50%**	Referent		--	--
** LVEF <50%**	1.10 (0.79–1.53)	0.59	--	--
**NYHA functional class, per class ^3^**	1.40 (1.05–1.82)	0.022	1.24 (0.91–1.69)	0.17
***De novo* heart failure vs other**	2.10 (1.29–3.43)	0.003	--	--
**Betablocker, yes vs no**	0.55 (0.38–0.79)	0.001	--	--
**ACE-, AT_1_R-inhibitors or ARNI, yes vs no**	0.38 (0.28–0.51)	<0.001	0.49 (0.35–0.70)	<0.001
**MRA, yes vs no**	1.13 (0.81–1.56)	0.48	--	--
**Amiodarone, yes vs no**	1.89 (1.12–3.19)	0.017	--	--
**Glycoside, yes vs no**	1.42 (1.01–2.00)	0.043	1.58 (1.10–2.27)	--
**Ivabradine, yes vs no**	0.67 (0.31–1.44)	0.30	--	--
**Admission heart rate, per tertile ^4^**	1.03 (0.85–1.24)	0.80		
**Discharge heart rate, per tertile ^5^**	1.22 (0.99–1.51)	0.06		
**R-mode stimulation**				
** No-CIED**	Referent			
** CIED-0**	1.10 (0.66–1.82)	0.72	0.97 (0.55–1.71)	0.92
** CIED-R**	2.61 (1.61–4.23)	<0.001	2.41 (1.42–4.10)	0.001
**Type of cardiac device**				
** No-CIED**	Referent		--	--
** Pacemaker**	1.43 (0.84–2.44)	0.19	--	--
** Implantable cardioverter defibrillator**	0.90 (0.40–2.05)	0.81	--	--
** Cardiac resynchronisation device**	2.24 (1.36–3.67)	0.001	--	--
**Type of cardiac device**				
** Pacemaker**	Referent			
** Defibrillator**	1.14 (0.60–2.18)	0.69	--	--

** Prevalence of any type of atrial fibrillation, assessed at discharge. ^1^ Tertiles of NT-proBNP were: T1 <284, T2 2848–7264, T3 >7264 pg/ml. ^2^ Tertiles of Charlson comorbidity index were: T1 ≤2, T2 3–4, T3 >4. ^3^ NYHA functional class II to IV, at admission. ^4^ Tertiles of heart rate at admission were: T1 <74 bpm, T2 74–86 bpm, T3 >86 bpm. ^5^ Tertiles of heart rate at discharge were: T1 <64 bpm, T2 64–76 bpm, T3 >76 bpm.

CIED = cardiac implantable electronic device, R = with rate response sensor (accelerometer), 0 = without rate response sensor (accelerometer), no-CIED = no CIED, NT-proBNP = N-terminal pro-hormone B-type natriuretic peptide, eGFR = estimated glomerular filtration rate, LVEF = left ventricular ejection fraction, ACEi = angiotensin converting enzyme inhibitor, ARB = angiotensin II receptor type 1 blocker, ARNI = angiotensin receptor neprilysin inhibitor, MRA = mineralocorticoid receptor antagonist, PM = pacemaker, ICD = implantable cardioverter defibrillator, CRT-D = cardiac resynchronisation device with defibrillator.

## Discussion

The current study investigated the effect of the presence of a CIED and an activated R-mode on the outcome of patients admitted with AHF. Two key observations were made: First, an increased resting heart rate at discharge was associated with a higher 12-month mortality risk only in patients *without* an activated R-mode. Second, an activated R-mode was associated with a consistently increased 12-month mortality risk compared to both, patients not carrying a CIED as well as CIED patients not dependent on R-mode stimulation. This association was independent of LVEF, type of CIED, burden of comorbidities, type of presentation (*de novo* vs. chronic) and betablocker therapy. Together, these findings show that an activated R-mode is associated with worse outcome in AHF and suggest that underlying chronotropic incompetence may not be adequately treated through accelerometer-based R-mode stimulation during and after an episode of AHF.

### Prognostic utility of resting heart rate in AHF

We observed a high risk of rehospitalisation or death of any cause in this vulnerable patient population, in line with earlier reports [14]. Our finding of a 36% increment in 12-month mortality risk per tertile of heart rate (measured at discharge) in AHF patients not dependent on R-mode simulation highlights that a lower heart rate prior to discharge, a marker of lower adrenergic drive, is a protective factor also in the setting of AHF. The SHIFT trial showed that heart rate reduction favourably affects mortality risk in patients suffering from chronic, stable HF with reduced LVEF [3]. As SHIFT comprised only few patients carrying CIEDs (CRT 1%, ICD 4%), it is unknown, whether the prognostic benefit of heart rate reduction may be generalized towards HF patients with CIEDs. The current study included a significant number of CIED patients and suggests that the protective effect of heart rate lowering could also apply to chronotropic competent carriers of CIEDs prior to discharge after AHF, hence in a recompensated state.

### Prognostic utility of an activated R-mode

To the best of our knowledge, this study is first to investigate the effect of R-mode activation on post-discharge mortality risk in AHF patients. As, according to current guidelines, frequency adaption (R-mode) is only indicated in the setting of compromised chronotropy, an activated R-mode can be regarded a surrogate for underlying chronotropic incompetence. The hypothesis that AHF patients suffering from chronotropic incompetence may have a worse prognosis compared to patients capable of intrinsic rate adaptation can be aligned with consequences of two important technical limitations of currently used sensors: i) The currently available sensor technology does not restore physiological rate adaption comparable to chronotropically competent patients [15]; ii) Accelerometers as the most widely used sensor type [12] are disadvantageous in resting states with a high metabolic demand, e.g. in the setting of AHF. Whereas accelerometers facilitate a rapid response to exercise, they fail to increase heart rate in the absence of physical activity. Therefore, when hospitalised for AHF, chronotropic incompetent patients carrying CIEDs with accelerometer based sensors may experience insufficient rate-adaptation leading to more severe hemodynamic derangement, which, importantly, is not only associated with acute substantial end-organ dysfunction but invariably leads to acute organ damage determining long-term prognosis [16]. Measurements of biomarkers related to cardiac injury as well as repeated evaluations of LV-function at the time of and after an AHF episode were shown to be useful to quantify AHF-related organ damage and predict mortality risk [16, 17]. In line with the hypothesis that insufficiently treated chronotropic incompetence aggravates organ damage during AHF, our data showed that patients carrying a CIED operating in rate-adaptive pacing mode were affected by increased 12-month mortality risk. Patients in group CIED-R showed the lowest heart rates at admission, the smallest decrement of heart rate during hospitalisation, and experienced a 12-month mortality approximately twice as high (51%) as observed in no-CIED (24%) or CIED-0 (27%). The markedly lower heart rate at admission observed in group CIED-R indicates clinically relevant chronotropic incompetence in these patients and contradicts concerns that an inappropriately activated R-mode may induce pathological heart rate elevation. As the increased mortality risk in CIED-R patients was also maintained after multivariable adjustment this argues against the hypothesis that a CIED *per se* mediates a worse prognosis. We rather postulate that the unfavourable hemodynamic derangement and the inability to adequately modulate cardiac output in chronotropic incompetent AHF patients further aggravates acute organ damage with subsequent negative long-term effects [16].

Interestingly, mortality of CIED-R carriers 12 months after hospital discharge was comparably high in patients with LVEF below and above 50%. This suggests that a blunted rate response to metabolic demands may be equally detrimental to both subtypes of HF. This is consistent with the fact that stroke volume cannot adequately be adapted in either subtype of HF, which increases the importance of rate adaptation to modulate cardiac output. Furthermore, the mortality risk observed in CIED-R patients did not depend on the type of device implanted (PM-R vs. ICD-R, 48% vs. 56%), which also emphasizes the strong negative impact of chronotropic incompetence on mortality risk in AHF patients irrespective of the type of CIED.

In accordance with these results, several studies identified chronotropic incompetence as a prognostic marker of adverse events in populations of chronic HF [4, 6]. In a sub-study of the HF-ACTION trial investigating 1118 chronic HFrEF patients with LVEF ≤35%, chronotropic incompetence was associated with an increased risk for mortality or rehospitalisation [6]. Another study in 1723 patients with chronic HF and LVEF ≤40% found an independent prognostic value of chronotropic incompetence in the subgroup of patients in sinus rhythm [4]. Taken together, chronotropic incompetence appears to strongly predict an adverse outcome in both acute and chronic HF.

### Chronotropic incompetence–innocent bystander or risk factor?

Despite several reports consistently showing an association of chronotropic incompetence and increased mortality risk in HF [2, 4, 6], there is still controversy, if reversal of chronotropic incompetence by rate-adaptive pacing may improve exercise capacity or prognosis [18–25]. On the one hand, chronotropic incompetence characterized by desensitization and downregulation of cardiac β-receptors due to chronic sympathetic overactivation [26] might be a sign of worsening HF. As such, it may not be the underlying cause of increased mortality, but a sign of advanced disease. In support of this hypothesis, an interventional long-term study with 77 HFrEF patients suffering from chronotropic incompetence showed that rate-adaptive pacing (DDDR, minute ventilation (MV) sensor technology) after 2CH-ICD implantation was inferior to VVI backup pacing with regard to clinical events and prognosis [24]. However, these results were limited by a crossover rate of 27% from VVI to DDDR-mode due to persistent bradycardia. Another more recent study including 10 HFrEF patients after 2CH-ICD implantation showed that atrial rate-adaptive pacing during treadmill testing resulted in a markedly increased peak heart rate, but was not accompanied by a corresponding increase in peak oxygen consumption if compared to stress testing in ventricular backup pacing-mode [23].

On the other hand, chronotropic incompetence also occurs in patients not suffering from HF demonstrating that its pathomechanism is not necessarily linked to progression of HF. Of note, HF patients with chronotropic incompetence have a limited possibility to adapt stroke volume and are thus at higher risk of organ malperfusion in states of metabolically increased demand. In line with this, several studies indicated positive effects of rate-adaptive pacing on exercise capacity or prognosis. In a small study in 20 patients with HFrEF carrying a CRT and suffering from severe chronotropic incompetence, rate-adaptive pacing increased peak heart rate and peak oxygen consumption compared to treadmill stress testing without rate-adaptive pacing [18]. This is consistent with a recent study demonstrating a positive effect of rate-adaptive pacing on walking distance during 6-minute walk test in 60 HFrEF patients with severe chronotropic incompetence [19]. Further, a positive impact of rate-adaptive pacing on prognosis in HF patients was shown in a sub-study from the LALTITUDE population including 1154 CRT-D patients. Here, rate-adaptive pacing using accelerometer technology was associated with better survival compared to matched patients without activated rate-adaptive pacing modes [25]. Further, it is also conceivable that neither chronotropic incompetence per se nor insufficient R-mode functionality are responsible for the increased mortality risk observed in CIED-R subgroup, but rather an increased rate of RV stimulation, which has repeatedly been shown to exert detrimental effects in HF. However, rate-adaptive pacing in dual chamber PMs primarily increases atrial pacing burden and does not necessarily translate into an increase of ventricular pacing. This is because a direct R-mode induced increase of RV stimulation is only typical for single chamber devices programmed in VVIR mode. The fact that the 12-month mortality risk remained virtually unaltered when restricting analyses to patients carrying a CRT or a CIED not programmed in VVIR-mode, argues against a major impact of R-mode induced increment of right ventricular pacing in this analysis.

Taken together, the conflicting evidence regarding the prognostic benefit of a reversal of chronotropic incompetence in HF may be the result of patient selection (definition and cut-off values of chronotropic incompetence), the amount of RA and RV-pacing and technical limitations regarding rate adaption.

### Technical solutions for rate adaption

Data from previous studies indicate that sensor technologies apart from accelerometers might be beneficial in HF patients. Dual sensor technologies with blended activity and MV sensors or closed-loop-stimulation (CLS) sensors allow improvement of device-dependent rate response profiles [12, 15, 27]. In the LIFE (Limiting Chronotropic Incompetence for Pacemaker Recipients) study, a better metabolic slope was achieved using dual sensor technology (accelerometer, MV) compared to an activity sensor alone [27]. The PROVIDE study compared CLS and accelerometer sensors in 131 patients with chronotropic incompetence carrying either a 1-CH-PM or 2-CH-PM and observed superior rate response of CLS compared to accelerometer sensors during mental stress testing [28].

### Implications for patient care and research

Overall, the strong association between an activated R-mode and increased mortality risk in our study population of patients admitted with AHF hints towards a potentially important modifiable treatment target with major relevance for patient care. In order to advance the knowledge base, further prospective studies including randomized controlled trials elucidating a possible causal link are needed. It should be investigated prospectively, if the excess mortality risk observed in our subgroup of CIED-R patients with accelerometer-based rate modulation can be confirmed in chronotropic incompetent AHF patients with i) CIEDs using CLS or MV technology and ii) CIEDs with temporarily deactivated R-mode stimulation. This trial could clarify, if insufficient rate adaptation or rate adaptive pacing itself is a clinically relevant harmful principle.

## Limitations

Our results should be considered in the light of several limitations: 1) Postulating an activated R-mode in CIED patients as a surrogate for chronotropic incompetence does not account for the potential presence of chronotropic incompetence in patients not carrying a CIED. Further, due to the retrospective analysis, we cannot determine specific diagnostic criteria for activation of rate-adaptive pacing in each case. Therefore, using an activated R-mode as a surrogate of chronotropic incompetence may not be justified in every individual patient if CIED-programming is not guideline-compliant. 2) The percentage of atrial and ventricular pacing as well as the daily timespan of R-mode activation remained unknown in a relevant proportion of patients. Although rate adaptive pacing does not necessarily translate into increased RV-stimulation, a potential negative impact of increased RV pacing on mortality risk in group CIED-R cannot be excluded. 3) It should be noted that the sample size of CIED patients included in the final analysis was modest and encompassed CIEDs from different manufacturers. 4) Further, group assignment (CIED-R, CIED-0, and no-CIED) is based on the status at admission (index hospitalisation); changes in group assignment during follow-up due to potential reprogramming and/or implantation of a CIED are not considered in this analysis. 5) In this study, we describe a statistically significant association between activation of rate adaptive pacing and an increased mortality risk which does not establish a cause-and-effect relation. 6) Differences regarding the dose of diuretics and further HF medication between the subgroups analysed were not reported. Therefore, an impact of these variables on the study’s results cannot be excluded.

## Supporting information

S1 File Institutional approach to evaluation of chronotropic incompetence in CIED carriers. (DOCX)

S1 Graphical abstract (TIFF)
