# Supplementary material for: Activated rate-response is associated with increased mortality risk in cardiac device carriers with acute heart failure
Source: PLoS One. 2024 Apr 18;19(4):e0302321. doi: 10.1371/journal.pone.0302321 (PMC11025974; doi:10.1371/journal.pone.0302321)
Supplement: S1 File — (DOCX) [file pone.0302321.s001.docx]

**Supplemental S1:** Institutional approach to evaluation of chronotropic incompetence in HF patients with CIEDs

In the first step, the patient's medical history is taken with a focus on physical activity in daily life assessing type and extent of physical exertion. Given physical activity, we analyse the distribution of the frequency histogram recorded by every CIED. Reduced or missing heart frequency modulation is taken as a first indicator for the presence of chronotropic incompetence(1). Additionally, increased atrial (DDD pacemaker, DDD-CRT) or ventricular pacing burden (VVI pacemaker in permanent atrial fibrillation) at the lower frequency limit further strengthens the likelihood for chronotropic incompetence. If available, additional clinical data (Holter ECG, smart watch recordings) are used for further assessment. In patients with no (bed-ridden) or very limited activities (mobility limited to the own apartment) the frequency histogram is either not or very cautiously evaluated. In case of doubt regarding the presence of chronotropic incompetence, treadmill testing is performed. Age predicted maximal heart rate (APMHR) in heart failure is assessed using the equation proposed by Brawner et al. (APMHR=119 + 0.5 x resting heart rate – 0.5 x age) (2). If all findings are suggestive of chronotropic incompetence, we activate rate-adaptive pacing and the effects (history, heart rate modulation, atrial and ventricular pacing burden) are re-evaluated after a few days in our device clinic. DDD pacemakers are programmed in accordance with best clinical practice to minimize an increase in ventricular pacing burden.

**References:**

1. Wilkoff BL, Richards M, Sharma A, Wold N, Jones P, Perschbacher D, et al. A Device Histogram-Based Simple Predictor of Mortality Risk in ICD and CRT-D Patients: The Heart Rate Score. Pacing Clin Electrophysiol. 2017;40(4):333-43.

2. Brawner CA, Ehrman JK, Schairer JR, Cao JJ, Keteyian SJ. Predicting maximum heart rate among patients with coronary heart disease receiving beta-adrenergic blockade therapy. Am Heart J. 2004;148(5):910-4.
