# Supplementary figures and images for: Activated rate-response is associated with increased mortality risk in cardiac device carriers with acute heart failure
Source: PLoS One. 2024 Apr 18;19(4):e0302321. doi: 10.1371/journal.pone.0302321 (PMC11025974; doi:10.1371/journal.pone.0302321)

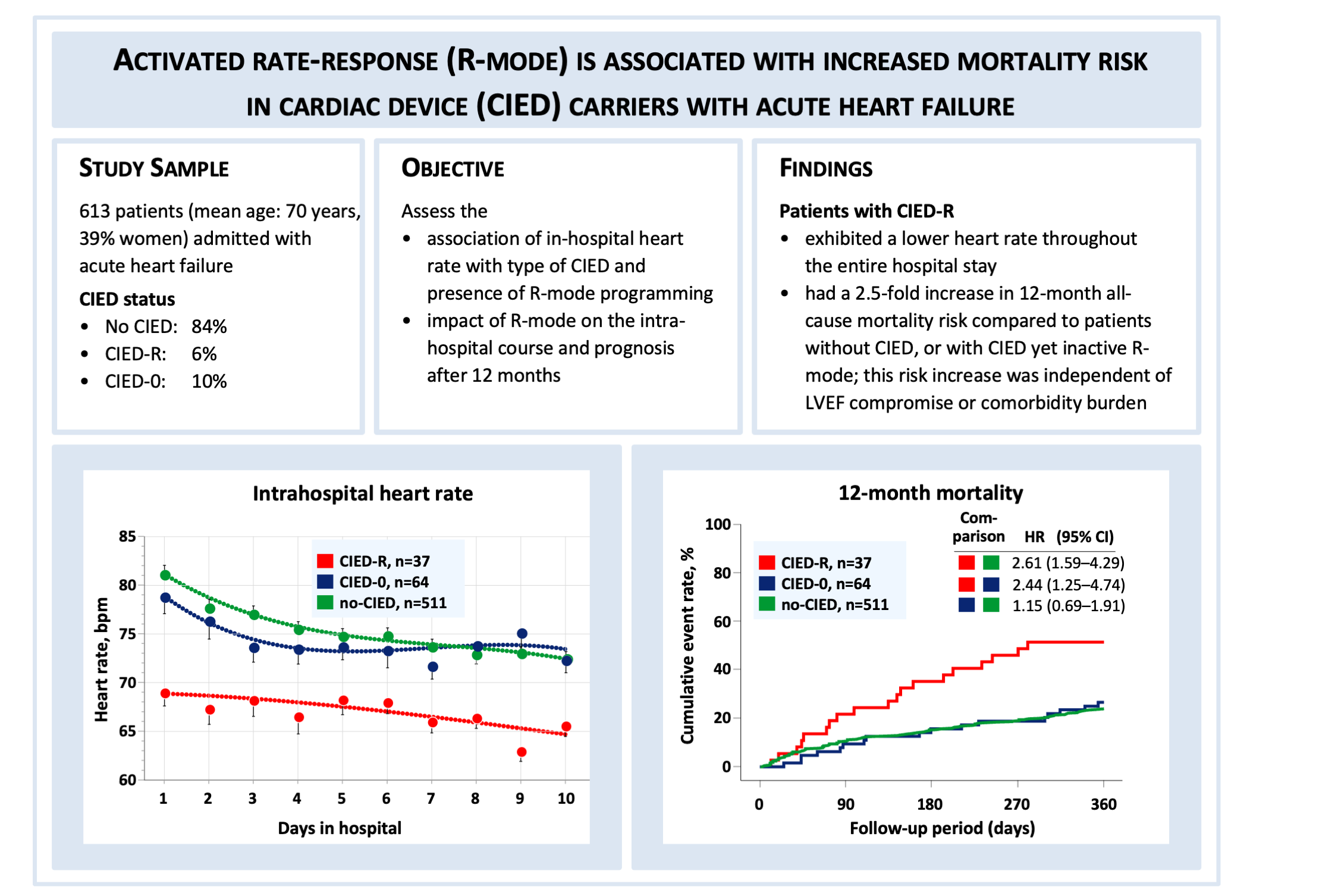

Supplement: S1 Graphical abstract — (TIFF) [file pone.0302321.s002.tiff]
